# Loading and Release of Charged and Neutral Fluorescent Dyes into and from Mesoporous Materials: A Key Role for Sensing Applications

**DOI:** 10.3390/mi12030249

**Published:** 2021-02-28

**Authors:** Estela Climent, Mandy Hecht, Knut Rurack

**Affiliations:** 1 Bundesanstalt für Materialforschung und Prüfung (BAM), Richard-Willstätter-Str. 11, 12489 Berlin, Germany; mandy.hecht@codecheck.info (M.H.); knut.rurack@bam.de (K.R.); 2 CodeCheck GmbH, Gneisenaustraße 115, 10961 Berlin, Germany

**Keywords:** mesoporous materials, charged dyes, neutral dyes, dye loading optimisation, dye release

## Abstract

The aim of this study is to determine the efficiency of loading and release of several zwitterionic, neutral, anionic and cationic dyes into/from mesoporous nanoparticles to find the optimum loading and release conditions for their application in detection protocols. The loading is carried out for MCM-41 type silica supports suspended in phosphate-buffered saline (PBS) buffer (pH 7.4) or in acetonitrile, involving the dyes (rhodamine B chloride, rhodamine 101 chloride, rhodamine 101 perchlorate, rhodamine 101 inner salt, *meso*-(4-hydroxyphenyl)-boron–dipyrromethene (BODIPY), sulforhodamine B sodium salt and fluorescein 27). As a general trend, rhodamine-based dyes are loaded with higher efficiency, when compared with BODIPY and fluorescein dyes. Between the rhodamine-based dyes, their charge and the solvent in which the loading process is carried out play important roles for the amount of cargo that can be loaded into the materials. The delivery experiments carried out in PBS buffer at pH 7.4 reveal for all the materials that anionic dyes are more efficiently released compared to their neutral or cationic counterparts. The overall best performance is achieved with the negatively charged sulforhodamine B dye in acetonitrile. This material also shows a high delivery degree in PBS buffer.

## 1. Introduction

The ability to control the release of cargo from porous materials has been largely associated with drug delivery and widely used in biomedical applications [1]. It is currently even employed to combat the viral SARS-CoV-2 pandemic [2]. However, in the last years the scope of this technology has been expanded toward applications in a variety of sectors such as agriculture [3,4], photovoltaic cells [5] external coatings [6] or personal care and cosmetics [7], employing as nanoparticles drug carriers (NPs) of organic [8,9,10] or inorganic [11] nature. Among these materials, microporous and mesoporous materials, due to their chemical inertness, homogeneous porosity and large internal surface area, have attracted considerable research interest for applications on the fields of drug delivery [12,13,14], catalysis [15,16,17], filtration and separation [18,19], gas adsorption [20,21] and storage [22,23], enzyme immobilisation [24,25], biomedical tissue regeneration [26,27], environmental remediation [28,29,30], chemical/biochemical sensing [31,32,33] and theranostics [34,35] mostly as nano- or microparticles, but also in core/shell formats or in combination with other properties such as magnetic ones [36,37]. Whereas typical microporous materials are crystalline framework solids, such as zeolites [38] with pore dimensions between 10–14 Å [39,40], mesoporous silica materials of the MCM-41 type, discovered in 1992 by researchers of the Mobil Research and Development Corporation [41,42], present a hexagonal arrangement of the mesopores, a homogeneous pore size of 2–3 nm, a high pore volume and specific surface areas of ca. 1000 m^2^ g^−1^ [43]. Due to these favourable features, these materials are ideal supports for the loading of (bio)molecular cargo into the pores and they have been extensively used as reservoirs for controlled release applications. Especially in the last decade, MCM-41-type silicas have been also used to prepare stimuli-responsive gated materials by the covalent grafting of organic compounds derivatised with silane moieties onto the surface [44]. These gated materials are able to transport selected (bio)molecules to specific locations [45] and allow for a controlled release of the cargo such as a drug or a reporter upon an external stimulus or in the presence of a target analyte [37]. Besides cargo stored in the pores of the support material, system design for stimuli-responsive release usually involves gatekeeper molecules that are coated on the outer surface of the carrier particles and interact with bulky entities such as (bio)macromolecules as caps, thus closing the pores. This so-called gating chemistry can be cleaved by the stimulus to which the system is responsive. In this case, the presence of one molecule of a target analyte is able to allow the release of hundreds of cargo molecules such as an optical or electrochemical reporter, because a much higher number of molecules can be stored in the inner of the pores of the support than analyte molecules are necessary to interact with the gating chemistry and induce the opening of a pore. The result is an intrinsic chemical signal amplification. These highly porous, container-like structures are thus particularly exciting vehicles for drug delivery [12] and chemical or biochemical sensing [46,47], in case of the latter especially in a lateral flow assay format [48,49].

For the design of a potent delivery or sensory system, important prerequisites are the loading and/or release efficiencies of the cargo molecules into and from the mesoporous material. The most widely used loading process is the steric incorporation of organic compounds that involves immersing the porous particles into a solution of the desired guest molecule, e.g., a fluorescent dye or a drug. The abundance of silanol groups (Si-OH) on the surface of the mesoporous MCM-41 silica particles makes them hydrophilic and thus dispersible in aqueous environments. If the interactions between the cargo and the particle (typically through hydrogen bonding or electrostatic interactions) in the system are favourable, the guest molecules move from the solution into the pores, interact with surface functional groups and, after removal of the solvent by evaporation, remain in the pores.

There are several studies published dealing with the factors that govern the loading of the pore network of mesoporous materials with selected drugs [50,51,52]. In previous work, the solvent (in which the loading process is carried out) [53,54], the morphology of the inorganic mesoporous support [52,55,56] and the chemical nature of the selected cargo (polarity, charge, presence of moieties able to give intermolecular non-covalent interactions) [57,58] have been studied as factors that control the loading process. Despite these intensive research efforts, still only a limited number of studies have been reported in which the transport of organic molecules that are different from drugs has been analysed [59,60,61]. However, when aiming at the development of gated indicator release systems for sensing applications, the requirements are fundamentally different to most drug release approaches. For drug release, the cargo should usually be delivered in a slow, but continuous fashion over a longer period of time. For sensing, on the other hand, release should be fast and proportionally quantitative in the presence of an analyte to provide the result of such a test within a few minutes at maximum; in the absence of an analyte, non-specific release should be at a minimum, ideally zero. For a sensitive response, a nearly complete pore loading with the selected reporter, or at least the highest possible, is mandatory.

**Figure 1:**
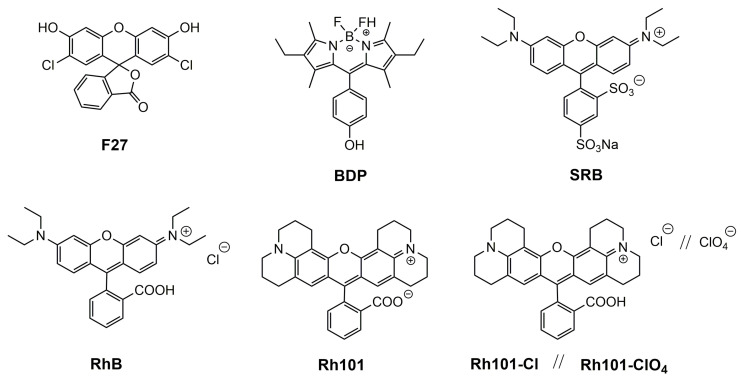
Chemical structures of selected dyes.

Our motivation for the work presented here was to determine the loading and release efficiencies of several neutral and differently charged dyes into and from mesoporous materials, fluorescent dyes being the favourable choice of reporter cargo as fluorescence commonly allows for the highest sensitivity in rather simple sensing formats. A MCM-41-type mesoporous solid was selected as inorganic scaffold; because such materials continue to be the most widely used ones, the dyes chosen were 2′,7′-dichlorofluorescein (**F27**) as neutral, rhodamine 101 inner salt (**Rh101**) and a boron–dipyrromethene (BODIPY) derivative (**BDP**) as zwitterionic, rhodamine B chloride (**RhB**), rhodamine 101 chloride (**Rh101-Cl**) and rhodamine 101 perchlorate (**Rh101-ClO_4_**) as cationic as well as sulforhodamine B (**SRB**) as anionic dye (Figure 1), all of them readily commercially available, and the two solvents employed were phosphate-buffered saline (PBS, at pH 7.4) and acetonitrile (MeCN).

## 2. Materials and Methods

### 2.1. Reagents

Chemicals and solvents were purchased from Sigma-Aldrich (Steinheim am Albuch, Germany), Merck (Darmstadt, Germany) and Fisher Scientific (Schwerte, Germany) in the highest quality available. The buffers and solutions were prepared with ultrapure reagent water, which was obtained by running demineralised water (by ion exchange) through a Milli-Q ultrapure water purification system Millipore Synthesis A10 from Merk (Darmstadt, Germany).

### 2.2. Buffer Solutions

Phosphate-buffered saline (PBS 10×; 70 mmol dm^−3^ Na_2_HPO_4_, 10 mmol dm^−3^ NaH_2_PO_4_, 145 mmol dm^−3^ NaCl, pH 7.4) was used for controlled guest molecule loading processes and release experiments.

### 2.3. Determination of Molar Absorption Coefficient of the Dyes

Each kind of fluorescent dye molecule absorbs maximally (*A_max_*) at a given wavelength (*λ_max_*) to an extent described by its molar absorption coefficient (*ε*), allowing for quantitation via the *Beer–Lambert* law [62] according to Equation (1):

(1) $$A=lg\frac{I_{0}}{I_{1}}=\varepsilon \cdot c\cdot d$$

where *A* corresponds to the absorbance of the sample, *I*_0_ is the intensity of light passing through the reference cell, *I*_1_ is the intensity of light passing through the sample cell, *ε* corresponds to the molar absorption coefficient of the absorber, *c* is the concentration of the absorbing species and *d* corresponds to the path length.

For the determination of the molar absorption coefficients, stock solutions of ca. 1 × 10^−4^ M of each dye were prepared in triplicate in acetonitrile and PBS, and these solutions were diluted to absorbance values between 0.1 and 1. In general, three measurements per replicate were performed.

### 2.4. Synthesis of Mesoporous MCM-41-Type Silica Nanoparticles (MSN)

*N*-cetyltrimethylammonium bromide (CTAB, 1.00 g, 2.74 mmol) was first dissolved in deionised water (480 mL) before adding NaOH (3.5 mL, 2.00 mol L^−1^) in deionised water, followed by adjusting the solution temperature to 80 °C. Tetraethoxysilane (TEOS, 5.00 mL, 25.7 mmol) was then added dropwise to the surfactant solution. The mixture was stirred for 2 h to give a white precipitate. The solid product was centrifuged, washed with deionised water and ethanol, and dried at 60 °C (MCM-41 as-synthesised). To prepare the final porous material (MCM-41), the as-synthesised solid was calcinated at 550 °C using an oxidant atmosphere for 5 h, thermally removing the template phase.

### 2.5. Materials Characterisation

Powder X-ray diffraction (PXRD), elemental analysis, transmission electron microscopy (TEM), N_2_ adsorption–desorption analysis and UV-visible absorption spectroscopy techniques were employed to characterise the synthesised materials and test their behaviour. PXRD measurements were performed on a Philips D8 Advance diffractometer (Bruker, Berlin, Germany) using CuKα radiation in the range from 1.3 to 10 2Θ. Elemental analyses were carried out with a Euro EA-Elementalanalysator (HEKAtech, Wegberg, Germany). TEM images were obtained with a 100 kV Philips CM10 microscope (Philips, Eindhoven, Neetherlands). For the TEM images, a drop of an aqueous suspension was placed on a copper grid and dried at room temperature. N_2_ adsorption–desorption isotherms were recorded with a Micromeritics ASAP2010 automated sorption analyser (Micrometrics, Unterschleissheim, Germany). The samples were degassed at 120 °C in vacuum overnight. The specific surface areas were calculated from the adsorption data in the low-pressure range using the Brunauer–Emmett–Teller (BET) model. The pore sizes were determined following the Barrett–Joyner–Halenda (BJH) method. UV-vis spectra were measured with a Specord 210plus from Analytik Jena (Jena, Germany).

### 2.6. Loading of Dyes into MCM-41

Solutions of the dyes were firstly prepared in acetonitrile and PBS. The concentration of the solutions was adjusted to 2 mmol L^−1^ for all cases, except for acetonitrile solutions of **Rh101** (1.6 mmol L^−1^) and **SRB** (1 mmol L^−1^), due to their lower solubility in acetonitrile. Then, mesoporous MCM-41 nanoparticles (10 mg) were suspended in acetonitrile or PBS solutions of the dyes, yielding suspensions with a final concentration of 0.8 mmol dye g^−1^ solid. The suspensions were stirred for 24 h at room temperature. Thereafter, each suspension was centrifuged (10 min at 6000 rpm) and dried using a vacuum dryer at 40 °C for 12 h, yielding the final solids. With this procedure, the residual solvent, which is adsorbed strongly to the pore walls, is removed. In general, the loading of the dyes into the MCM-41 materials was repeated two times.

### 2.7. Quantification of Dye Loaded

UV–visible absorption measurements were carried out to determine the amount of dye loaded into the materials. Furthermore, elemental analyses were used to confirm the dye contents obtained through UV–visible measurements. In an indirect approach, we measured the concentration of each dye in the aqueous solutions before and after the loading process through absorbance measurements at their lowest-energy absorption maximum (*A_max_*) and analysis via the *Beer–Lambert* law. On the other hand, the amount of dye loaded into the materials was assessed directly via elemental analysis (C, N, S), revealing the amount of dye in mmol per gram of SiO_2_ (mmol g^−1^ SiO_2_) using Equation (2):

(2) $$a_{A}=\frac{\Delta W_{i}\%\;x\;1000}{\;\Delta W_{SiO_{2}}\%\;x\;nM_{i}}\;\;\;\left(\frac{mmol}{g\;SiO_{2}}\right)$$

where Δ*W_i_*% (*i* = C, N, S) are the weight percentages of carbon, nitrogen or sulphur, Δ*W_SiO_*_2_% is the inorganic SiO_2_ content in weight percentage, *M_i_* is the corresponding atomic weight and *n* is the number of the corresponding atom type in one molecule. In general, two measurements were performed for each replicate of material.

### 2.8. Dye Release Experiments

Because the aim of the work was to find the optimum loading and release conditions for mesoporous materials to be used in sensing applications, release experiments were performed in PBS buffer at pH 7.4. Moreover, because sensing aims at fast responses, only short release times <5 min were evaluated. The release of dyes from all the solids was carried out by suspending each material (1 mg) in PBS (2 mL) while stirring with a vortex for 30 s before centrifugation and measurement of the absorption spectra of the corresponding solutions. Additionally, kinetic release experiments were carried out by suspending the correspondent solid (0.3 mg) in PBS (3 mL) and registering the absorbance of the released dye over time. In general, release experiments from MCM-41 materials were repeated two times.

## 3. Results

### 3.1. Characterisation of Silica Mesoporous Nanoparticles

**Figure 2:**
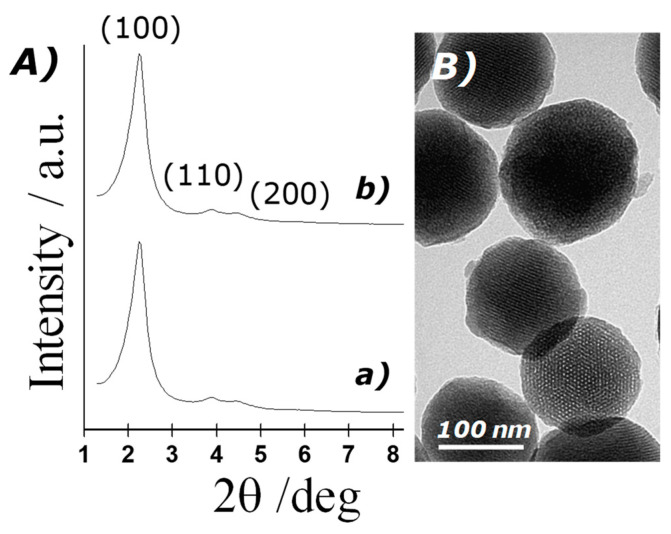
(**A**) Powder X-ray patterns of the solids: (**a**) MCM-41 as-synthesised and (**b**) calcinated MCM-41; (**B**) TEM images of calcinated MCM-41, showing the typical hexagonal porosity of the mesoporous matrix.

The structure of the MSN prepared was confirmed by powder X-ray diffraction (PXRD), transmission electron microscopy and N_2_ adsorption–desorption studies. Figure 2A shows PXRD patterns of the solids MCM-41 as-synthesised and MCM-41 calcinated. The PXRD of nanoparticulated siliceous MCM-41 as-synthesised (curve a) shows the typical low-angle reflections that can be attributed to a hexagonal array, denoted as (100), (110) and (200) Bragg peaks, and from the XRD data of MCM-41 as-synthesised, a d_100_ spacing of 42.39 Å was calculated. PXRD of the calcinated MCM-41 sample (curve b) shows a significant displacement of the (100) peak corresponding to an approximate cell contraction of 2.89 Å, due to the further condensation of silanol groups during the calcination step. PXRD measurements of loaded solids were not carried out due to the low amount of material prepared. However, a further reduction in the intensity of d_100_ reflexion is expected because of dye loading. Transmission electron microscopy (TEM) analysis also confirmed the mesoporous structure of MSN, showing spherical particles with diameters from 80 to 120 nm and the typical hexagonal porosity and channels of the MCM-41 matrix as alternating black and white stripes (see Figure 2B).

**Figure 3:**
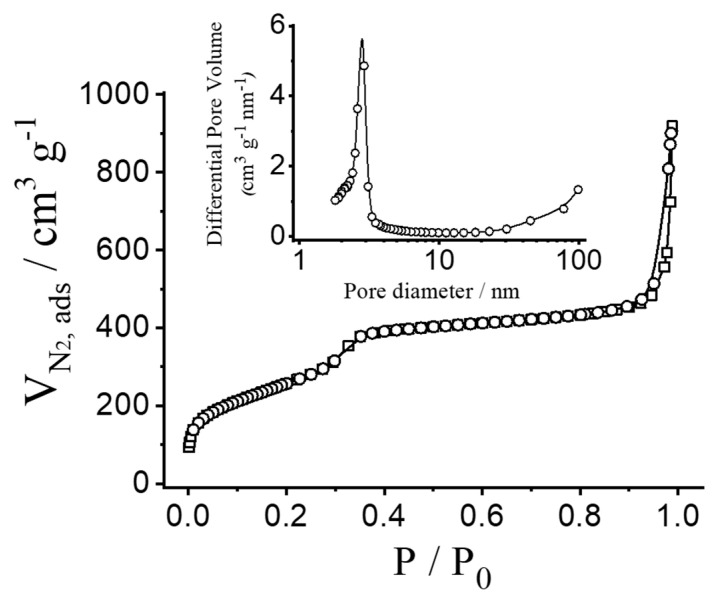
N_2_ adsorption–desorption isotherms for calcinated MCM-41 nanoparticles. Inset: pore size distribution of MCM-41 nanoparticles.

To quantify the porous nature of the MSN, N_2_ adsorption–desorption isotherms were measured and analysed. Figure 3 shows N_2_ adsorption–desorption isotherms of the calcinated nanoparticulated MCM-41 material. This curve corresponds to a type IV isotherm, in which the observed step is related to nitrogen condensation inside the mesopores, with an adsorption step at intermediate pressure *P*/*P*_0_ values (0.25–0.4). The application of the BET model resulted in a value of 937.6 m^2^ g^−1^ for the total specific surface area, a pore diameter of 2.75 nm and a pore volume of 0.75 cm^3^ g^−1^. From the PXRD, porosimetry and TEM studies, the a_0_ cell parameter (4.56 nm), the pore diameter (2.75 nm) and the value for the wall thickness (1.81 nm) were calculated. The isotherm also shows another adsorption step at high relative pressure (*P*/*P*_0_ > 0.85), which is associated to the filling of the large voids among the particles (pore diameter of 40.11 nm and pore volume of 0.51 cm^3^ g^−1^, calculated by using the BJH model) and which must therefore be considered as textural porosity. The existence of uniform cylindrical mesopores is suggested by the absence of a hysteresis loop at intermediate *P*/*P*_0_ values (0.25–0.4) and also from the narrow BJH pore distribution. N_2_ adsorption–desorption isotherm measurements of the loaded particles were not carried out because of the high amount of material that would be necessary. However, a further reduction in the specific surface area and pore volume is expected as a consequence of dye entrapment in the porous network.

### 3.2. Selection of Dyes and Loading Procedure

The sensitivity of a gated sensing material is mainly influenced by the loading/adsorption step as well as the releasing/desorption process of the reporters stored in the voids. To gain insight into these processes in more detail and to obtain optimised materials for best possible performance, several common organic dyes (cationic, anionic, neutral and zwitterionic) were selected: 2′,7′-dichlorofluorescein (**F27**, neutral), rhodamine 101 inner salt (**Rh101**) and a BODIPY derivative (**BDP**), both zwitterionic, rhodamine B chloride (**RhB**), rhodamine 101 chloride (**Rh101-Cl**) and rhodamine 101 perchlorate (**Rh101-ClO_4_**), all cationic, and sulforhodamine B (**SRB**), anionic (Figure 1). These dyes were chosen because they are highly fluorescent under the conditions that are commonly used for lateral flow assays, i.e., aqueous samples, frequently buffered at (near-)neutral pH when biomacromolecules are used for gating, because they comply with the excitation sources and emission filter sets of most standard instrumentation and because they are widely commercially available [48,49]. While release is thus critically dependent on the behaviour of the system in aqueous media, loading can be performed in both aqueous and organic media. The different charge states and counterions determine the solubility of the dyes and their interaction with the silica surface, thus influencing the amount of cargo that can be stored as well as the release kinetics and efficiency.

For the loading of the mesoporous silica nanoparticles (MSN), acetonitrile was selected as an aprotic polar organic solvent with high dielectric constant, usually dissolving neutral and zwitterionic dyes as well as dyes which incorporate a dimensionally larger, delocalised organic ionic unit such as aminoxantheniminium, and phosphate-buffered saline at pH 7.4 as aqueous medium, being identical to common assay conditions. The combination of the seven dyes and the two solvents used for loading yielded the 14 materials listed in Table 1.

The loaded materials were obtained by suspending 10 mg of MCM-41 nanoparticles in acetonitrile or in PBS (4 mL) containing the respective dye at a concentration of 0.8 mmol g^−1^ solid, while stirring for 24 h. The efficiency of loading was then determined by UV-visible absorption measurements, using the molar absorption coefficients of the dyes (Table 2) and the *Beer-Lambert* law for quantitation.

**Figure 4:**
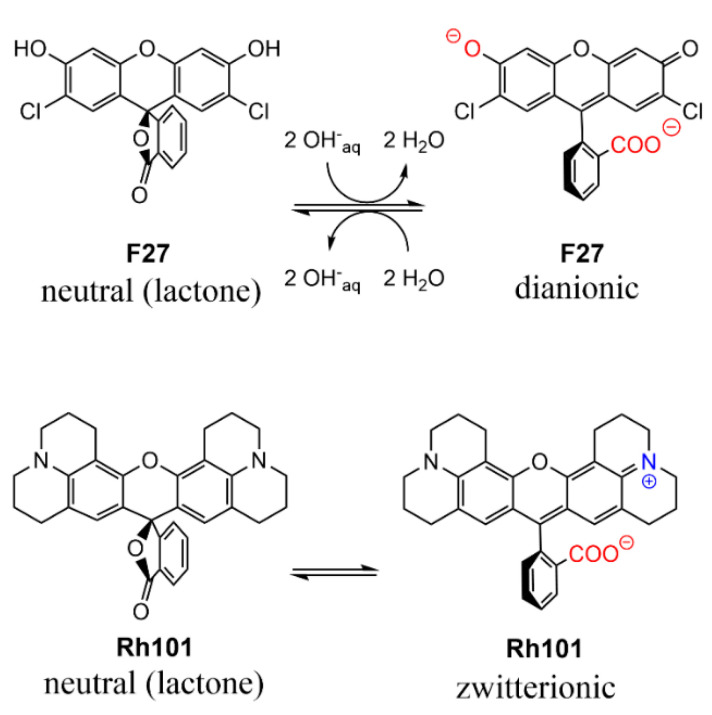
Prevalent charge states of the dyes in water at pH 7.4. Positively charged moieties are highlighted in blue and the negatively charged moieties are highlighted in red.

**Figure 5:**
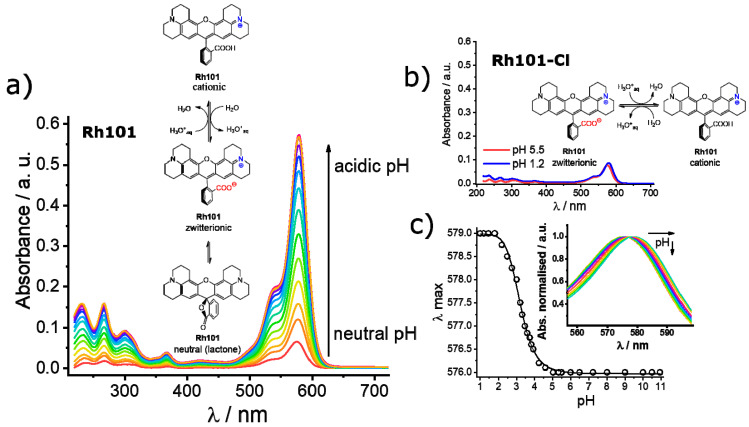
Absorbance registered upon pH titration of rhodamine 101 inner salt (**Rh101**) (**a**) and rhodamine 101 chloride (**Rh101-Cl**) (**b**) in water. Inset: conversion equilibria of the different forms from the neutral and/or zwitterionic form to the cationic form under acidic pH values. (**c**) Maximum of wavelength registered as a function of the pH for the **Rh101-Cl** in water. Inset: zoomed normalised absorption titration spectra of **Rh101-Cl**, showing the bathochromic displacement of the maximum of the band from 576 to 579 nm.

As can be seen in Table 2, the molar absorption coefficients of the dyes were slightly higher in PBS than in MeCN. Furthermore, the values found were, in most cases, in good agreement with values reported in the literature, e.g., 107,000 M^−1^ cm^−1^ for **RhB** in EtOH, 111,000 M^−1^ cm^−1^ for **SRB** in EtOH; 95,000 M^−1^ cm^−1^ for **Rh101-ClO_4_** in acidic EtOH and 110,000 M^−1^ cm^−1^ for **F27** in basic EtOH [63]. The lower molar absorption coefficient of the inner salt **Rh101** in both solvents in comparison with **Rh101-Cl** and **Rh101-ClO_4_** indicates that here the **Rh101** dye was most likely present as a mixture of its zwitterion and neutral (lactone) form in the commercial product purchased [64]. The same effect was observed for the dye **F27** in MeCN (see Figure 4 for the corresponding chemical structures). To demonstrate this, pH titrations of the dyes **Rh101** and **Rh101-Cl** were performed in H_2_O, see Figure 5a,b. In both cases, a small bathochromic shift was observed from neutral (576 nm) to acidic pH values (579 nm), yielding a p*K*_a_ of 3.2 (see Figure 5c). However, whereas the absorbance of the dye remained practically constant during the **Rh101-Cl** titrations (Figure 5b), indicating the conversion from the zwitterionic form to the cationic form after protonation, a huge increase in the absorbance was observed for the **Rh101** inner salt solution (Figure 5b). Closer inspection of the titration spectra reveals that, first, until pH ~ 4, the lactone form (only absorbing in the UV) was progressively converted into the zwitterionic form (absorbing in the visible), before the protonation-related equilibrium set in, leading to the shift that is characteristic for the cationic form.

### 3.3. Loading and Release Studies

The amounts of dye loaded into all the prepared materials in acetonitrile and in PBS are listed in Table 3. The load of the 14 prepared materials was determined directly by elemental analysis (EA) and indirectly by UV/vis absorption measurements of the supernatants. The results are presented in absolute terms (mmol g^−1^ SiO_2_) and in relative terms (average %). Table 3 also includes the release efficiency (in %) of the dyes delivered from each of the loaded materials in PBS at pH 7.4. To recall, the important factors for the discussion of the results are: (i) a possible relationship between loading efficiency, the charge state of a dye and the solvent; (ii) the role of the counter-ions and (iii) a possible correlation of these features for loading and release.

As a general trend, the relative loading efficiencies shown in Table 3 suggest that rhodamine dyes can be loaded much better into an MCM-41 scaffold (loading rates of 11–90%) than **F27** or **BDP** (loading rates of 8–15%), irrespective of the solvent used. The low loadings obtained with **F27** and **BDP** might be related to their lower hydrophilicity, at least when **F27** is in its neutral form and because **BDP** is the least hydrophilic dye used here. Focusing on all the rhodamines tested, we observed that the solvent and the charge state of the dye play an important role for the loading efficiency. Whereas cationic rhodamines (**Rh101-Cl**, **Rh101-ClO_4_** and **RhB**) were loaded in higher amounts when PBS was used as solvent instead of acetonitrile, the reverse behaviour (higher loading efficiency in acetonitrile than in PBS) was observed for the anionic (**SRB**) or zwitterionic (**Rh101**) rhodamines. In addition, the size and charge density of the counter-ion has also a decisive effect when comparing the behaviour of **Rh101-Cl** and **Rh101-ClO_4_** in both solvents. In the presence of the chloride anion, significantly more Rh101 can be incorporated than for the perchlorate anion.

## 4. Discussion

The loading/adsorption as well release/desorption ability of the cargo molecules is based on complex adsorption/desorption processes. Due to this, apart from general solubility (dye in solvent) and wetting (dispersibility, solvent filling of MCM-41 pores), the interactions between the cargo molecules, the solvent and the surface of the material need to be considered:

(i) Dye and solvent: the solvation process of the dye is mainly affected by dipole interactions and polarizability properties, depending on dipole moments and charge delocalisation. In addition, if a counter-ion is present, the equilibrium between well-solvated dye ion (D)_S_ and well-solvated counter-ion (I)_S_, a solvated ternary complex of a dye, a solvent molecule and a counter-ion (D•S•I)_S_ as well as a solvated yet tightly bound ion pair (D•I)_S_ has to be considered as a consequence of the electrostatic forces at play. These effects can stabilise the dissolution of the compound and reduce the adsorption ability of the dye within the material. On the other hand, a low solubility in the bulk solvent can enhance the adsorption into the pores of a material because of the favoured affinity between the surface functional groups and dye molecule.
(ii) Dye and dye: hydrophobic interactions such as π stacking between two dye molecules can influence the loading and release process.
(iii) Dye and silica surface: a satisfactory degree of loading can only be achieved if the interactions of the dye and the material are sufficiently strong. However, too strong interactions are not favoured because they would hinder a fast desorption process.
(iv) Silica surface: the surface pH of the porous material can affect the dissolution of the dye as a function of its own charge state, which is connected to the solvent’s bulk pH. Furthermore, the pore size and shape may have an influence on the mass transfer as well [65].

**Figure 6:**
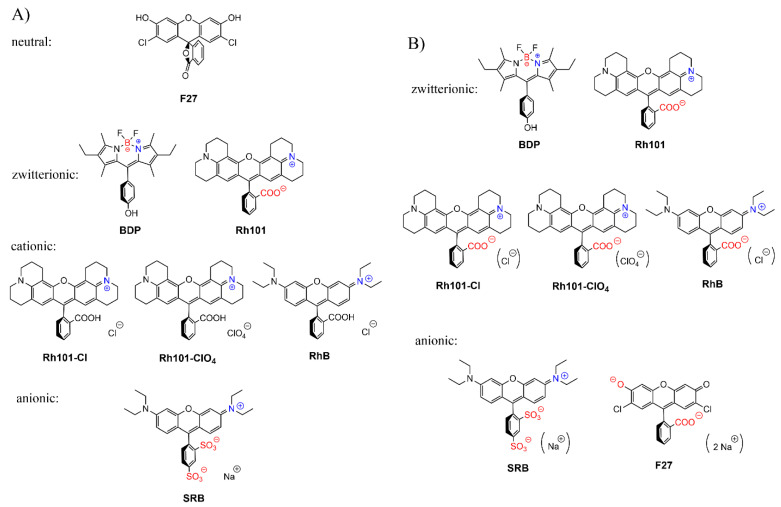
Prevalent charge states of the dyes in (**A**) acetonitrile and (**B**) phosphate-buffered saline (PBS) buffer at pH 7.4. Positively charged moieties are highlighted in blue, negatively charged in red; brackets around counter-ions in (**B**) denote that these species are well-solvated in PBS and are not involved in ion-pairing.

The charge state of the dyes is differently influenced by the two solvents used in this work. While the rhodamine dyes carry a permanently cationic aminoxantheniminium unit, their carboxylic acid groups are undissociated in acetonitrile, but will be anionic in PBS; p*K*_a_(**RhB**) = 3.7 [66], p*K*_a_(**Rh101**) = 3.2 (see previous section, Figure 5c). **Rh101** and **BDP** (p*K*_a_ = 9.9 for phenol group [67]) are zwitterionic in both solvents; **SRB**, employed as sodium salt, is net anionic in both solvents (p*K*_a_ ~ 0.5 for doubly sulfonated dyes [68]). **F27** is neutral in acetonitrile yet exists in the dianionic form in PBS at pH 7.4, because of its p*K*_a_ = 5.0 for the monanion–dianion equilibrium [69]. Moreover, whereas the charge centres of zwitterionic **BDP** are in close proximity, endowing the dye with an overall rather lipophilic, neutral character, those in **Rh101**—and in **Rh101-Cl**, **Rh101-ClO_4_** and **RhB** in PBS—are rather distant and located on perpendicularly-oriented molecular fragments. Among the latter, i.e., **Rh101** salts and **RhB**, the delocalised positive charge is better shielded by the bulkier julolidyl groups in the **Rh101** series. Figure 6 collects the prevalent forms of the dyes in the two solvents.

On the other hand, the choice of solvent also controls to a certain extent the charge state and chemical nature of the MSN support that has an immediate impact on the polarity inside (and on the outer surface) of the porous network. In PBS at pH 7.4, the surface of MSN has a net negative charge due to the presence of deprotonated silanol groups at the pore walls. Thus, repulsive electrostatic interactions between negatively charged dyes (for instance **SRB**) and the negatively charged pore walls can play a major role in the loading process. The data in Table 3 suggest that, in PBS at pH 7.4, positively charged dyes are preferentially loaded into the pores, followed by overall charge-neutral dyes with negatively charged dyes being the least preferred. Whereas the loading efficiency hardly exceeds 10% for the anionic dyes **SRB** and **F27**, it increases to 36.5% upon introduction of a positively charged functional group at a sufficiently distant part of the molecule as in zwitterionic **Rh101**. For **BDP**, with its closely situated zwitterionic sites, the low efficiency of ca. 14% suggests that polarity effects dominate, and no pronounced adsorption is effective. For the zwitterionic rhodamine dyes coming in the form of a binary salt, favourable uptake rates of 50% for **Rh101-ClO_4_**, 69% for **RhB** and 73% for **Rh101-Cl** are found, respectively. Here, the less charge-dense and more lipophilic perchlorate counter-ion seems to aggravate uptake to a certain degree as both dyes that can be most efficiently loaded, **Rh-101-Cl** and **RhB**, come with a Cl^−^ anion.

The results obtained in PBS suggest that the loading of dyes into mesoporous matrices is primarily governed by the chemical interaction between silanol groups covering the silica surface and the functional groups of the dye. Therefore, the specific surface area is expected to determine the amount of dye that can be incorporated into the silica matrix. Because electrostatic interactions are stronger in aqueous environments, the loading efficiency of the cationic dyes is generally higher in PBS than in acetonitrile. Such a behaviour is supposedly much less pronounced for intrinsically zwitterionic compounds. Favourable electrostatic effects on drug loading have been reported in the literature before. For instance, Balas and co-workers loaded alendronate, a potent (anionic) bisphosphonate used in osteoporosis treatments, into MCM-41 (specific surface area of 1157 m^2^ g^−1^) and SBA-15 (specific surface area of 719 m^2^ g^−1^) mesoporous matrices and found that under the same conditions, the maximum amounts of drug loaded were 14% and 8% for MCM-41 and SBA-15, respectively [70]. To increase the attractive host–guest interactions between alendronate and support, Balas et al. functionalised both supports with amino groups and achieved generally more efficient loading in both cases, yet the trend among the two materials followed that of the unmodified materials, i.e., alendronate could be loaded at 37% into amino-MCM-41, but only 22% into amino-SBA-15. This difference was accordingly attributed to the higher surface area of MCM-41. On the other hand, this work also showed that electrostatic matching—anionic phosphonate groups on the drug vs. anionic silica surface or cationic ammonium-functionalised silica surface—can increase the storage efficiency by a factor of three.

This scenario changes completely when acetonitrile was used for the loading process instead of PBS in our present work. Whereas in PBS the polarity of the environment outside and inside of the mesopores is rather similar, it is distinctly different in acetonitrile. Because of the considerably narrow pore diameter and the presence of silanol groups on the walls of the mesopores, the polarity in the pore voids is expected to be higher than in the bulk acetonitrile phase. Accordingly, differences in solubilities of the dyes in both solvents can influence the loading rates to a larger extent. If the solubilities of the dyes are considered, it is evident that, whereas the positively charged dyes **Rh101-Cl**, **Rh101-ClO_4_** and **RhB** can be easily dissolved at 2 mM or higher in acetonitrile, this amount is limited to 1 and 1.6 mM for **SRB** and **Rh101**; however, no such limitations exist for PBS. The reversed loading behaviour of **SRB** and **Rh101** vs. **Rh101-Cl**, **Rh101-ClO_4_** and **RhB**, i.e., higher loading in MeCN vs. PBS for **SRB** and **Rh101** yet higher loading in PBS vs. MeCN for the other three, see Table 3, suggests that polarity-controlled solubility issues promote the transit of **SRB** and **Rh101** from the bulk phase into the pores. This driving force is higher for **SRB** (eight-fold better loading in MeCN than in PBS) than for **Rh101** (two-fold), reflecting their dissolution behaviour.

**Figure 7:**
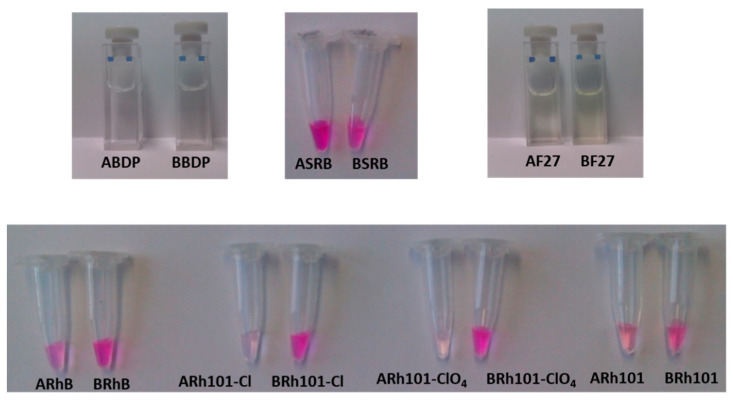
Photographs of released dye in the supernatant after centrifugation of suspensions of the 14 materials.

Having identified the forces that determine the loading efficiency, it is essential to analyse the release or delivery processes, because the overall performance of drug delivery or gated indicator release systems is determined by both features. As written above, in view of the fields of application such materials are used in, the delivery studies for the 14 materials were carried out only in PBS buffer at pH 7.4. The data in Table 3 reveal a general trend, i.e., that the materials loaded in PBS are able to deliver more cargo than their counterparts prepared in acetonitrile, see Figure 7 for better illustration. This finding is tentatively attributed to the different degrees of solvation of the dyes in both solvents. Poorer solvation in acetonitrile presumably leads to stronger interactions with the silanol/silanolate groups located at the pore walls, aggravating desorption from the wall and diffusion out of the pores in PBS. On the contrary, better solvation of the partners in PBS leads to reduced interactions between dyes and pore walls, facilitating exiting in PBS. Keeping in mind that after loading the materials have to be dried, see Section 2.6, the differences in wetting behaviour of the materials prepared in PBS and MeCN might also play a role, but were not investigated further here, because they are less relevant from an applied point of view. The trends regarding the loading/release ability can be summarised as follows:

A-materials: **BDP** < **F27** < **SRB** < **Rh101-ClO_4_** < **Rh101-Cl** ~ **Rh101** < **RhB**
B-materials: **BDP** < **Rh101-ClO_4_** < **Rh101-Cl** < **F27** < **RhB** < **Rh101** < **SRB**

An anionic dye such as **SRB** is generally more efficiently delivered (73% and 61% release) when compared with cationic (3–28% release), neutral and zwitterionic (0–35% release) dyes. Despite the higher release observed for **BSRB** (73% that corresponds to 0.0657 mmol dye g^−1^ SiO_2_), the utility of this material in controlled release protocols is hampered by the low amount of dye loaded (11%) when compared for instance with that of **ASRB** (90% loading and 61% release, corresponding to an absolute release of 0.4363 mmol dye g^−1^ SiO_2_). In accordance with the general trend, the delivery efficiency of materials containing a cationic dye cargo that had been loaded in PBS (**BRh101-Cl**, **BRh101-ClO_4_** and **BRhB**) is higher than that of materials that have been loaded in acetonitrile.

The more efficient release of anionic dyes, when compared with their cationic counterparts, is presumably related to the polarity and charge of the mesoporous pore walls. The presence of negatively charged silanolate groups at the inner walls of the porous network leads to repulsive interactions with the negatively charged dyes, allowing for a faster and more quantitative release see, e.g., the release of **SRB** from **BSRB** and **ASRB**. The same effect was observed for the materials loaded with **F27** (**BF27** and **AF27**). When **F27** was loaded in PBS at pH 7.4, the dye exists mainly in its open, doubly deprotonated form and shows a loading efficiency that is very similar to that of **SRB**. However, when acetonitrile was used as medium for loading, the dye is in its neutral lactone form. As for the other least hydrophilic dye, **BDP**, loading is also not efficient for this combination. Whereas for **BDP** the absence of interactive forces leads to low loading rates in both solvents, for **F27** the absence of forces for the neutral form and the presence of repulsive forces in the dianionic form seem to have an overall similar effect, i.e., loading efficiencies of only 12% and 15% in PBS and acetonitrile, respectively. This change in chemical nature is reflected by the release studies. Whereas for **BDP** release is virtually absent, release of neutral **F27** is also only low from **AF27** (17%), despite the use of a solvent that promotes anion formation, yet distinctly higher from **BF27** (45%) that incorporates primarily the open, dianionic form. The latter behaviour agrees well with the behaviour of **SRB**.

**Figure 8:**
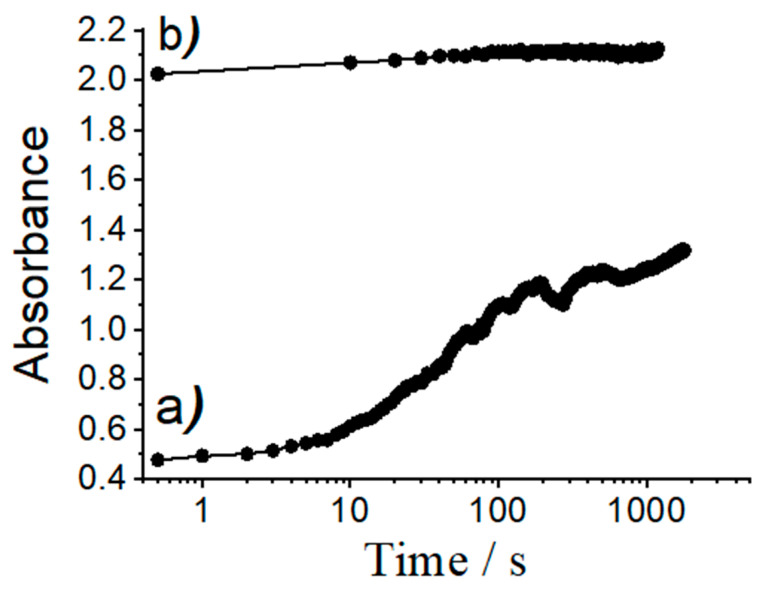
Absorbance values of dye released from solids (**a**) **ARhB** and (**b**) **ASRB** vs. time. Experiments were carried out by suspending ca. 0.3 mg of the corresponding materials in 3 mL of PBS in a quartz cuvette and monitoring dye release by measuring the absorbance values at 564 nm for **SRB** and at 555 nm for **RhB** vs. time.

On the other hand, attractive electrostatic interactions between the positively charged dyes (**RhB**, **Rh101-Cl** and **Rh101-ClO_4_**) and the negatively charged silanolate groups on the pore walls of the inorganic support are responsible for the generally low delivery observed, ranging from 3% to 28% of the total amount of dye loaded; this value is also only slightly higher for the inner salt **Rh101** with well-separated anionic and cationic fragments. In addition, it is important to note that the differences in release as a function of the solvent used for loading are rather similar for **RhB**, **Rh101-Cl** and **Rh101-ClO_4_**. Moreover, when loading and release are carried out in PBS, all three rhodamine 101 dyes behave very similar, i.e., ca. 13% of the initially used dye can be released. In addition to factors such as tighter or looser interaction of cargo molecules with functional groups of the pore surface, concentration effects might also play a role here. As Li and co-workers have observed, high loading rates can lead to lower release rates, presumably due to trapping and hindered diffusion in the voids [59]. These authors studied the loading of an MCM-41 scaffold with a neutral Hoechst dye at concentrations of 1 mM and 10 mM in the loading solution and then capped the pores with a supramolecular nanovalve. They found that, for the material loaded from the 1 mM solution, most of the dye molecules were eventually released from the particles upon opening of the nanovalve. However, the material loaded from the more concentrated dye solution was able to release only ca. 50% of its cargo. The authors concluded that particles loaded with the more concentrated solution yielded a material in which the dye molecules were more densely packed, limiting mobility during the release process; note that silica is a very rigid matrix. In addition, if dye molecules are tightly adsorbed to the inner pore walls, diffusion within the 1D channels would be hindered, reducing the release efficiency. The degree of such an effect would thus depend on the number of molecules adsorbed to the pore walls, in essence like a ball that has to find its way through a lane of a pin-ball machine that contains more or less obstacles. In line with our results, the authors also observed that the mobility of the cargo molecules was also affected by their electrostatic interaction with the support, i.e., that less tightly adsorbed molecules were more easily released. Bearing these facts in mind, we studied the release kinetics of an anionic (**SRB**) and a cationic (**RhB**) dye from materials **ASRB** and **ARhB**, see Figure 8. As could be seen, the release of the anionic dye from **ASRB** is virtually instantaneous, whereas the release of cationic **RhB** is significantly slower. The fast release observed for **SRB** is thus attributed to a process governed by electrostatic repulsion between the anionic dye and the negatively charged pore walls. Cationic **RhB** on the other hand is more strongly adsorbed to the pore walls, slowing down release kinetics.

Apart from electrostatic interactions, van der Waals forces can also play an important role in the loading and delivery processes. In fact, it has been demonstrated recently that such local interactions of van der Waals type between the porous host and guest molecules can modulate the macroscopic transport from the pores to the solution [71,72]. These authors concluded that stronger guest–matrix attractions (through van der Waals interactions) produced slower release kinetics and retained a higher amount of guest in the pores. Van der Waals interactions between guest molecules and the inorganic scaffold can lead to an accumulation of the former at the pore walls, which can be understood as a multilayer adsorption process, negatively affecting desorption (especially in a solvent like PBS) and diffusion. We assume that the negligible release rates for **BDP** from both materials **ABDP** and **BBDP** are presumably due to such non-polar interactions between the rather hydrophobic dye and the pore walls. However, such a preferential localisation of molecules at surfaces can also be facilitated by electrostatic interactions as Ng et al. have shown in loading and release studies of two fluorescent dyes (one anionic and one cationic) into and from mesoporous silica spheres by confocal laser scanning microscopy [73]. Analysis of the time-dependent release of both dyes showed clear differences: whereas the concentration profiles of the anionic dye within the spheres showed a homogeneous distribution and the release followed a simple diffusion-driven process, the concentration of the cationic dye was not homogeneous. In fact, the concentration of the cationic dye close to the surface of the spheres was higher compared to that in the core, and the release was controlled by slow diffusion after an initial process of rapid release.

Based on our experimental results and observations published previously by others, several conclusions can be drawn. (i) To achieve a high loading of cationic dyes into MSN materials (between 50% and 70%) aqueous media such as PBS buffer are the solvent of choice. (ii) In contrast, acetonitrile should be preferentially used for anionic dyes (e.g., 90% loading of **SRB**). (iii) Regarding the release process, which will be bound to aqueous media in most application cases whether drug delivery or sensing is required, the best results can be obtained with anionic dyes (60–75% release); cationic molecules apparently rather strongly interact with the pore walls, allowing for only low releases of 3–30%. The best performance was obtained with **ASRB**, i.e., with the anionic dye sulforhodamine B loaded in acetonitrile into the MCM-41 scaffold, because it allows storage of as much as 0.68 mmol **SRB** g^−1^ SiO_2_ (ca. 90% loading) and is able to release ca. 61% (0.4363 mmol g^−1^ SiO_2_) of the entrapped cargo in a desired application.

## 5. Conclusions

The loading of a dye into a mesoporous support is of crucial importance in the preparation of gated hybrid materials for their application in controlled release and recognition processes. Especially for analytical purposes, the preparation of loaded materials in which the cargo release is massive and fast is of crucial importance to achieve high levels of sensitivity. Our present study of 14 different materials, varying the chemical nature of the dye and the solvent for loading the cargo into the pores of an MCM-41 type silica material has revealed that the different charge states of the cargo molecule and the solvation ability of the solvent have the strongest impact, with counter-ion effects and non-polar interactions also contributing to success. Among all the dyes used here, the highest loading efficiencies were achieved with rhodamine derivatives (**Rh101**, **Rh101-Cl**, **Rh101-ClO_4_**, **RhB** and **SRB**). The content of cationic rhodamines (**Rh101-Cl**, **Rh101-ClO_4_** and **RhB**), which are actually zwitterionic under the respective conditions, is the highest when PBS was used as solvent. When using acetonitrile, anionic (**SRB**) and intrinsically zwitterionic (**Rh101**) rhodamines are loaded in higher amounts. The delivery experiments carried out in PBS buffer demonstrated that anionic dyes are more efficiently released compared with cationic dyes, due to repulsive interactions between dye and silica wall facilitating desorption. Neutral or rather hydrophobic dyes such as **BDP** show inferior performance, singling out **SRB** as the best suited dye when loading is carried out in acetonitrile. Our present studies suggest that a tremendous potential in the design of such release systems lies with the chemical tailoring of the cargo molecules as well as the surface tailoring of the walls of mesoporous silica materials. Besides introducing charged groups such as sulfonate groups to organic molecules, especially for hydrophobic molecules also the introduction of solubilising poly(ethylene glycol) groups can be considered [74]. The modularity and almost limitless ways of system optimisation become obvious when one considers that also the size and structure of the porous network can be altered in many different ways [75]. As a conclusion, all aspects of the composite system, i.e., the selection of the inorganic support, the selection of the dye and the choice of the solvent used for loading are essential to finally approach a hybrid (gated) mesoporous material with optimal performance for sensing (or drug delivery).

## Figures and Tables

**Table 1:** Overview of the loaded materials obtained by the various combinations of dye and solvent used for loading of the MCM-41 scaffold.

Solvent				Dyes			
	Rh101	Rh101-Cl	Rh101-ClO_4_	RhB	SRB	F27	BDP
MeCN	**ARh101**	**ARh101-Cl**	**ARh101-ClO_4_**	**ARhB**	**ASRB**	**AF27**	**ABDP**
PBS	**BRh101**	**BRh101-Cl**	**BRh101-ClO_4_**	**BRhB**	**BSRB**	**BF27**	**BBDP**

**Table 2:** Molar absorption coefficients (M^−1^ cm^−1^) of dyes in acetonitrile and PBS buffer at the corresponding absorption maxima.

Dye	Solvent	λ_max_ (nm)	ε_max_^a^ (M^−1^ cm^−1^)
**Rh101**	PBS	576	44580
**Rh101**	MeCN	576	12690
**Rh101-Cl**	PBS	576	86630
**Rh101-Cl**	MeCN	576	86330
**Rh101-ClO_4_**	PBS	576	96890
**Rh101-ClO_4_**	MeCN	572	96160
**SRB**	PBS	564	95950
**SRB**	MeCN	550	73320
**RhB**	PBS	555	97520
**RhB**	MeCN	554	87120
**F27**	PBS	503	92510
**F27**	MeCN	503	300
**BDP**	PBS	- ^b^	- ^b^
**BDP**	MeCN	507	69950

^a^ Uncertainties of measurement for *ε*_max_ ≤ ±5%, except ± 10% for **F27** in MeCN. ^b^ Not determined because of low solubility.

**Table 3:** Amount of dye (mmol g^−1^ SiO_2_) loaded into and delivered from the various materials.

Material	mmol g^−1^ SiO_2_ Loaded; via EA	mmol g^−^ SiO_2_ Loaded; via UV	mmol g^−^ SiO_2_ Delivered	% Loaded (Average) *^a^*	% Delivered
**BRh101**	0.229 ± 0.015	0.354 ± 0.035	0.103 ± 0.001	36.5	35.2
**ARh101**	0.577 ± 0.040	0.658 ± 0.045	0.156 ± 0.015	77.2	25.3
**BRh101-Cl**	0.559 ± 0.089	0.603 ± 0.053	0.103 ± 0.013	72.6	17.8
**ARh101-Cl**	0.229 ± 0.008	0.091 ± 0.038	0.005 ± 0.001	20.0	3.0
**BRh101-ClO_4_**	0.501 ± 0.067	0.299 ± 0.005	0.096 ± 0.007	50.0	23.9
**ARh101-ClO_4_**	0.177 ± 0.007	0.111 ± 0.011	0.004 ± 0.001	18.0	2.7
**BSRB**	0.097 ± 0.104	0.082 ± 0.001	0.066 ± 0.003	11.2	73.1
**ASRB**	0.756 ± 0.029	0.682 ± 0.002	0.436 ± 0.011	89.9	60.7
**BRhB**	0.467 ± 0.034	0.631 ± 0.002	0.156 ± 0.002	68.6	28.3
**ARhB**	0.300 ± 0.017	0.533 ± 0.028	0.037 ± 0.005	52.1	8.9
**BF27**	0.091 ± 0.012	0.0980 ± 0.020	0.042 ± 0.005	11.8	44.6
**AF27**	0.115 ± 0.035	0.1230 ± 0.030	0.0200 ± 0.002	14.9	16.8
**BBDP**	0.130 ± 0.002	-	-	13.8	-
**ABDP**	0.071 ± 0.010	0.0621 ± 0.045	0.006 ± 0.010	8.3	-

*^a^* Average from EA and UV/vis measurements, thus no error given.
